# Glaucoma related Proteomic Alterations in Human Retina Samples

**DOI:** 10.1038/srep29759

**Published:** 2016-07-18

**Authors:** Sebastian Funke, Natarajan Perumal, Sabine Beck, Silke Gabel-Scheurich, Carsten Schmelter, Julia Teister, Claudia Gerbig, Oliver W. Gramlich, Norbert Pfeiffer, Franz H. Grus

**Affiliations:** 1 Experimental Ophthalmology, Department of Ophthalmology, University Medical Center, Johannes Gutenberg University, Mainz, Germany; 2 Department of Ophthalmology and Visual Sciences, University of Iowa, Iowa, USA

## Abstract

Glaucoma related proteomic changes have been documented in cell and animal models. However, proteomic studies investigating on human retina samples are still rare. In the present work, retina samples of glaucoma and non-glaucoma control donors have been examined by a state-of-the-art mass spectrometry (MS) workflow to uncover glaucoma related proteomic changes. More than 600 proteins could be identified with high confidence (FDR < 1%) in human retina samples. Distinct proteomic changes have been observed in 10% of proteins encircling mitochondrial and nucleus species. Numerous proteins showed a significant glaucoma related level change (p < 0.05) or distinct tendency of alteration (p < 0.1). Candidates were documented to be involved in cellular development, stress and cell death. Increase of stress related proteins and decrease of new glaucoma related candidates, ADP/ATP translocase 3 (ANT3), PC4 and SRFS1-interacting protein 1 (DFS70) and methyl-CpG-binding protein 2 (MeCp2) could be documented by MS. Moreover, candidates could be validated by Accurate Inclusion Mass Screening (AIMS) and immunostaining and supported for the retinal ganglion cell layer (GCL) by laser capture microdissection (LCM) in porcine and human eye cryosections. The workflow allowed a detailed view into the human retina proteome highlighting new molecular players ANT3, DFS70 and MeCp2 associated to glaucoma.

Glaucoma is a neurodegenerative ocular disease complex with multifactorial etiology displaying a characteristic damage pattern in the optic nerve head manifested by a progressive loss of retinal ganglion cells (RGCs), their axons and visual field^1^,^2^. In 2010, worldwide 60.5 million people suffered from glaucoma and over 70 million people are expected to develop glaucoma by 2020 making glaucoma to one of the leading causes of blindness affecting people of all ages, however with increasing prevalence with age^3^. Inflammatory^4^ and autoimmune processes^5^,^6^ implementing oxidative stress^7^ and mitochondrial dysfunction^8^,^9^ have been documented for glaucoma so far and underlying molecular mechanisms are shifting in focus of research. Accordingly, numerous studies have demonstrated proteomic changes referring to experimental *in-vivo* and *in-vitro* models, regarding rodents^10^,^11^,^12^ and primates^13^. Glaucoma related proteomic alterations have also been reported for human sample material, e.g. aqueous humor^14^,^15^,^16^,^17^,^18^,^19^, trabecular meshwork^20^,^21^ and tears^22^ proposing key proteins and biomarker candidates. Focusing on human glaucomatous retina samples, Tezel *et al*. documented hemoglobin upregulation using immunohistochemistry^23^, whereas the research groups of Yang and Luo revealed proteomic changes linked to TNF-α/TNFR1^24^ and Toll-like receptors (TLRs)^25^ using mass spectrometry. However, proteomic investigations on human retinal sample species are rare due to limitation of donors and human sample material. Therefore, the purpose of the present study was to analyze human retinal samples of glaucoma (*N* = 5) and non-glaucoma subjects (*N* = 5) by use of a state-of-the-art “bottom-up” high performance liquid chromatography electro spray ionization mass spectrometry (BU LC ESI MS) workflow. Retinal sample exploration should provide an in-depth view to the human retina proteome, reveal glaucomatous proteomic alterations and should contribute to a better understanding of the molecular pathomechanism of glaucoma. Moreover, new molecular candidates linked to glaucomatous neurodegeneration should be proposed giving direction for future glaucoma research projects.

## Results

**Figure 1:**
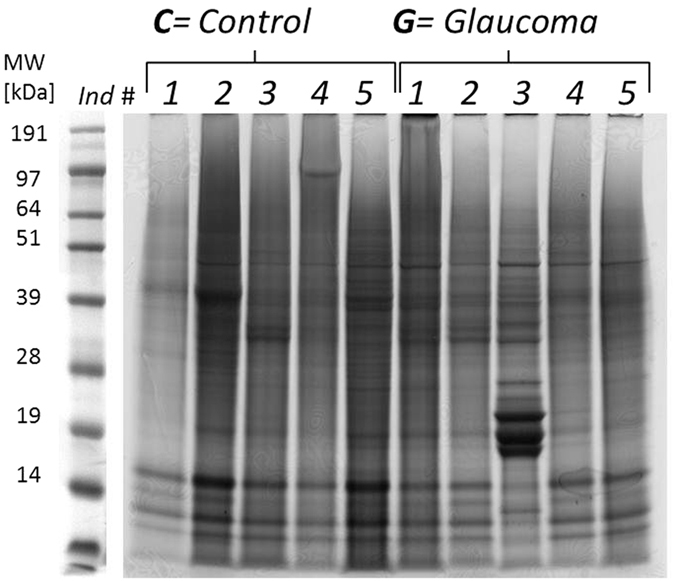
1D SDS PAGE pattern of individual retina lysate samples of glaucoma and control donors (*N* = 5/group). Sample lanes, containing each 80 μg of total protein, have been equally divided into 17 slices following tryptic in-gel digestion. Sample #G3 was excluded from later statistical analysis due to a unique protein cluster between ~20 and ~15 kDa.

**Figure 2:**
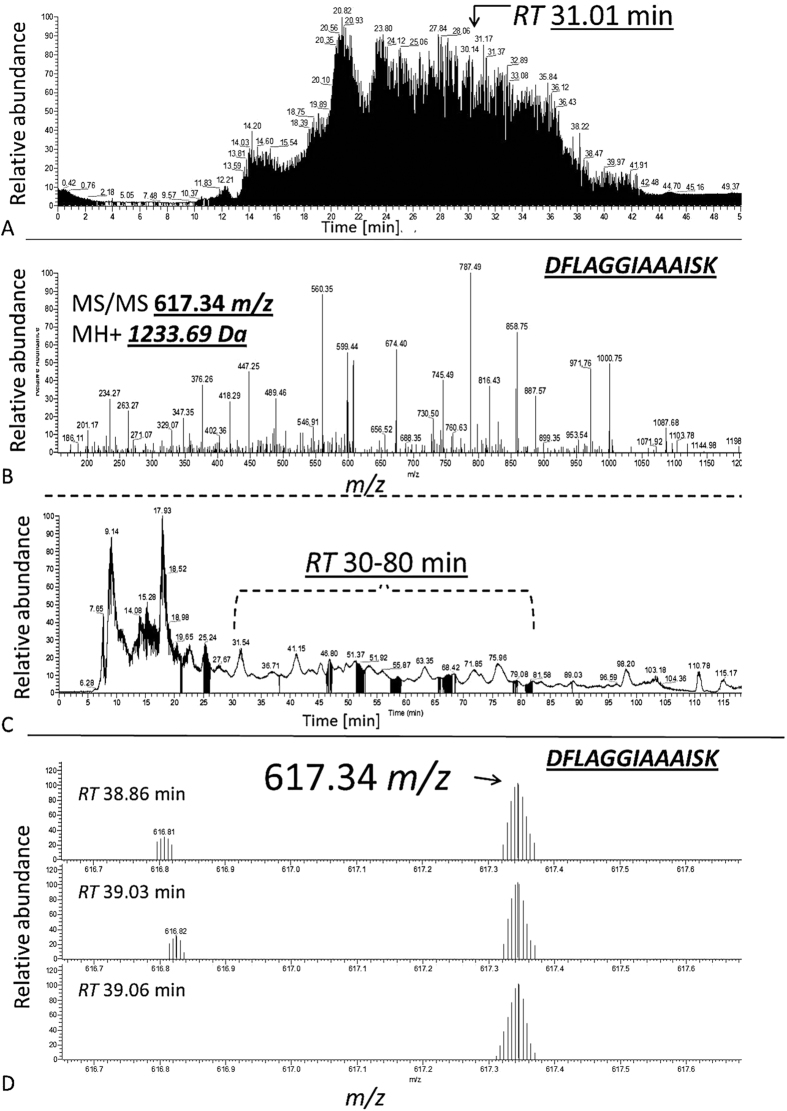
(**A**) Exemplary LC ESI MS TIC chromatogram of a single gel slice peptide extract corresponding to a control retina lysate highlighting the exemplary identification of ANT3 at *RT* 31.01 min. within the 50 min gradient (**B**). MS/MS fragment spectrum of 617.34 m/z (2+; MH+ 1233.69 Da) contributing to the identification of ANT3 (**C**). Elution range of the unique ANT3 reporter peak at 617.34 m/z between *RT* 30–80 min corresponding to an in-solution digest of a control retina lysate pool (**D**). Exemplary MS spectra at three *RT*s showing the ANT3 reporter peak 617.34 m/z corresponding to the amino acid sequence: *DFLAGGIAAAISK*.

**Figure 3:**
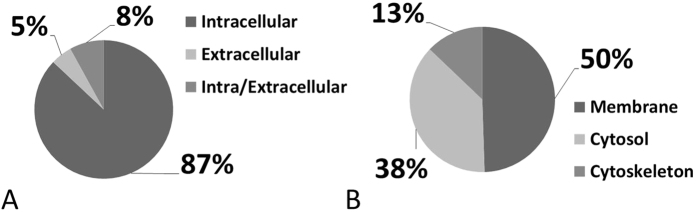
Cellular component distribution of human retinal proteins displaying glaucoma related level alterations. (**A**) The majority of affected proteins are intracellular species, representing predominantely (**B**) membrane proteins.

**Figure 4:**
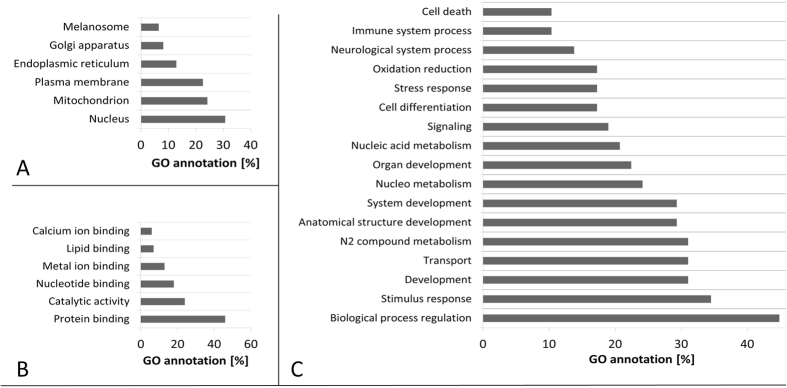
Localization and functional GO analysis of human retinal proteins showing glaucoma related level changes. (**A**) Predominantly, nucleus and mitochondrial proteins showed level changes associated to glaucoma. (**B**) Candidates display binding and catalytic properties. (**C**) A high portion of candidates are involved in regulatory biological processes encircling developmental, transport processes and cell death.

**Figure 5:**
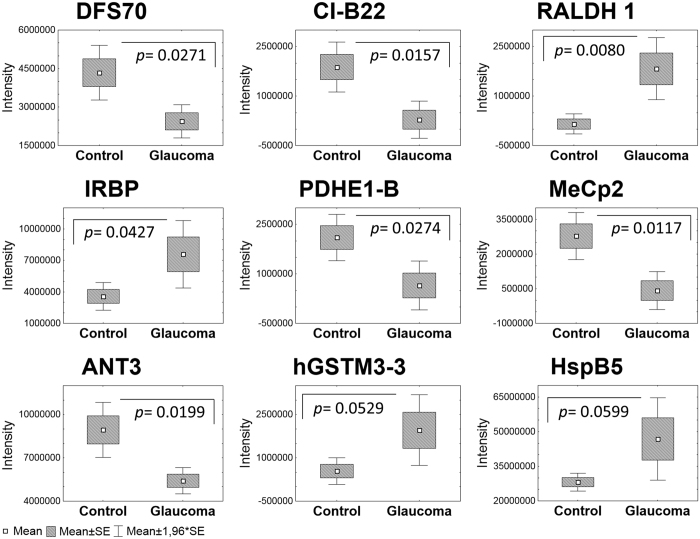
Exemplary human retinal proteins showing significant differences (p < 0.05) to distinct alteration tendencies (p < 0.1) in protein levels revealed by BU LC ESI MS following label-free quantification and t-test.

**Figure 6:**
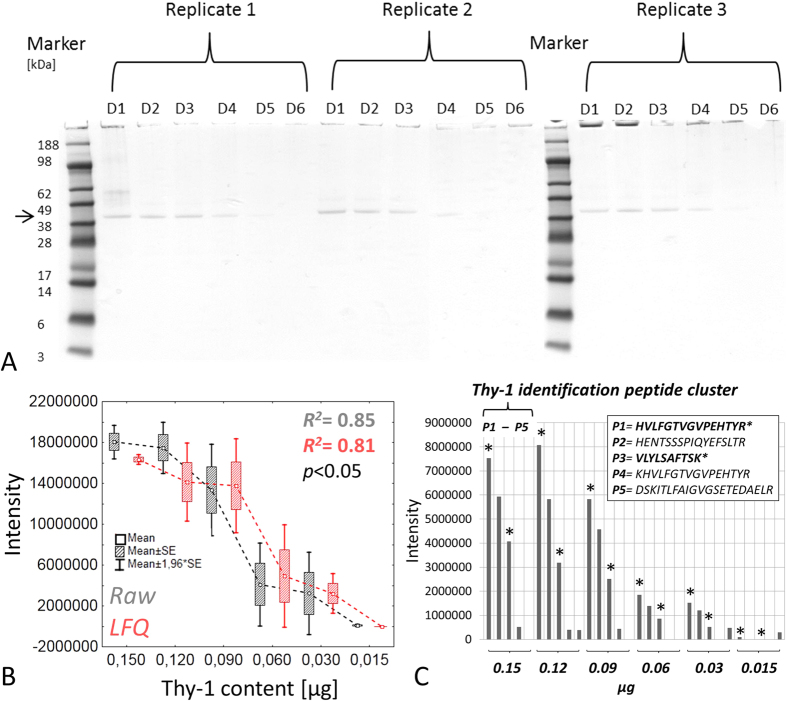
Dynamic MS detection range of recombinant Thy-1. (**A**) Recombinant Thy-1 1D SDS PAGE dilution experiment (43.34 kDa, mass migration area highlighted by arrow; D1 = 0.3, D2 = 0.24, D3 = 0.18, D4 = 0.12, D5 = 0.06, D6 = 0.03 μg; N = 3 replicate runs). Thy-1 content <0.12 μg is not visible by CBB staining. (**B**) Dynamic MS range of recombinant Thy-1 (50% of gel content is introduced to the LC MS system for technical reasons; therefore the X-axis displays corrected values) generated by MaxQuant software LC MS detection threshold can be determined <0.03 μg describing intensity values < 1*E^6^, whereby the LFQ value is calculated zero. (**C**). Separate evaluation of the Thy-1 specific peptides (P1–P5) indicating that for Thy-1 content <0.03 μg only two Thy-1 specific peptides can be detected (P1 & 3; highlighted by asterisk); which represent MS/MS identification peptides in study sample runs. Since in sample runs only two of five characteristic peptides (P1 & P3) could be detected showing raw output values <1*E^6^, Thy-1 can be attributed to the low detection range and is consequently underrepresented in the retina samples leading to critical quantification output.

**Figure 7:**
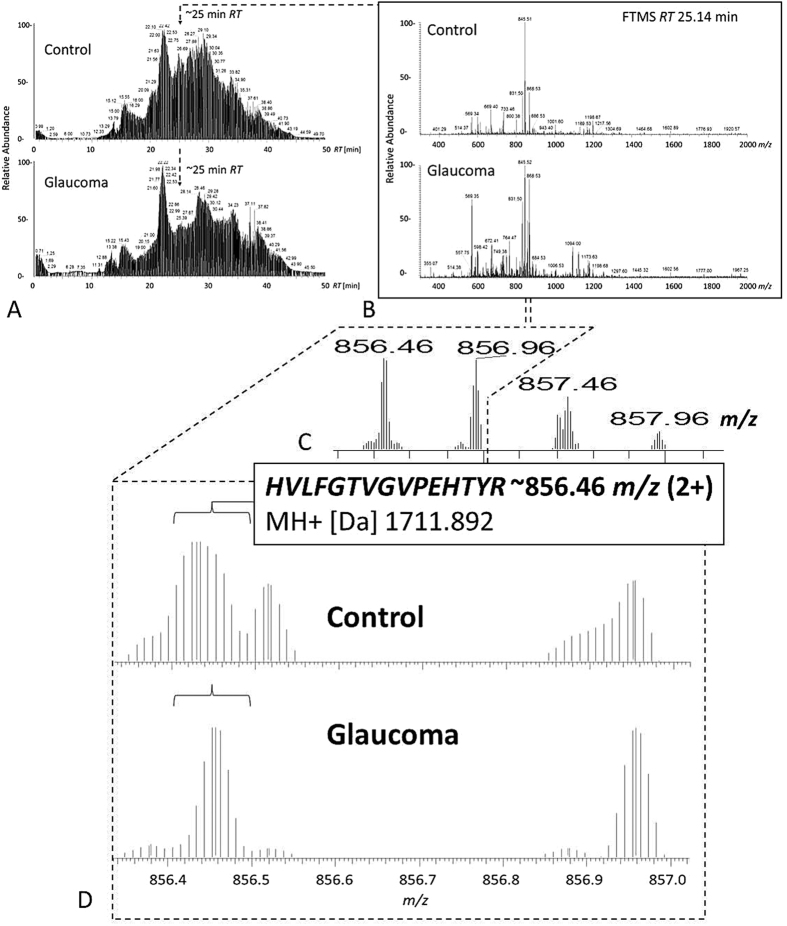
MS detection of Thy-1 in pooled retina samples (glaucoma *vs*. control). (**A,B**) Thy-1 could be identified in pooled glaucoma samples by MS/MS of 856.46 m/z. The corresponding peak is also detectable in the identical RT range at ~25 min in the control pool, however not leading to identification. (**C**) Isotopic pattern of Thy-1 specific peptide showing monoisotopic peak at ~856.46 *m/z.* (**D**). Zoom view to Thy-1 specific peptide isotopic pattern corresponding to MS spectrum of control and glaucoma pool sample supporting Thy-1 presence for both groups.

**Figure 8:**
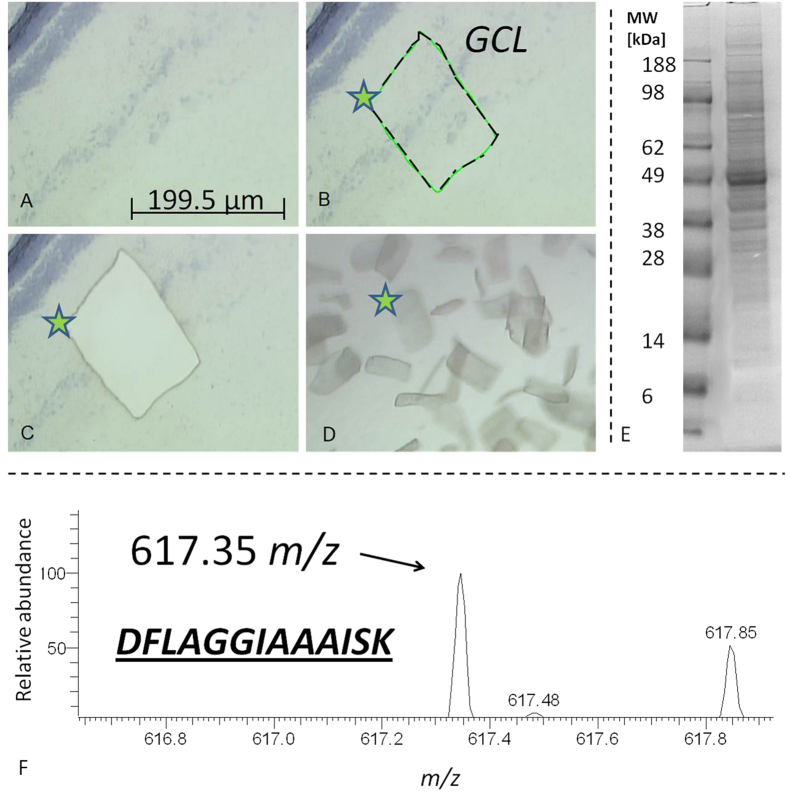
(**A–C**) Laser capture microdissection (LCM) of ganglion cell layer (GCL) tissue areas (indicated by asterisk) of porcine retina cryosections. (**D**) Exemplary GCL exudates. (**E**) 1D-SDS-PAGE Protein pattern of a GCL exudate extract pool destinated for BU LC ESI MS analysis. (**F**) Exemplary recovery of ANT3 in GCL exudates indicated by ANT3 unique reporter peak 617.35 m/z corresponding to the amino acid sequence *DFLAGGIAAAISK*.

**Figure 9:**
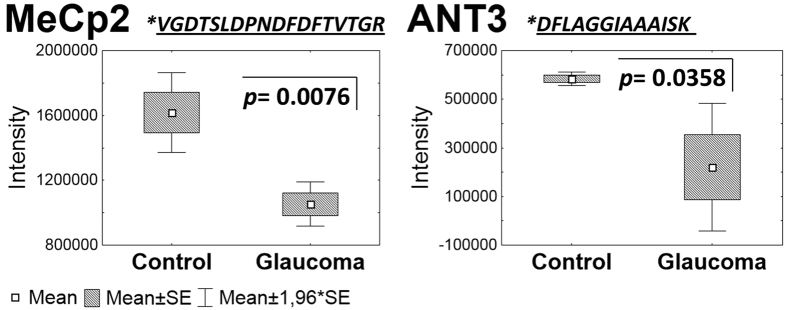
AIMS validation of selected retinal protein candidates: MeCp2 and ANT3 are decreased in pooled glaucoma samples (*N* = 4 individuals/group) supported by technical replicate runs (*N* = 4). Unique reporter peptide sequences are highlighted by asterisk.

**Figure 10:**
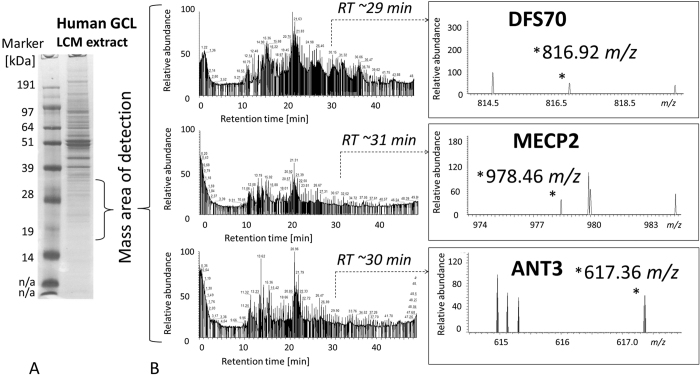
Detection of unique reporter peaks (indicated by asterisks in the MS spectra in B) corresponding to the three marker candidates DFS70, MECP2 and ANT3 in the retinal ganglion cell layer (GCL) of a human ophthalmic LCM preparation following (**A**). 1D SDS PAGE and (**B**). LC MS analysis. The preparation was based on 30 μm cryosections of a female non-glaucoma donor bulbus of a 69 year old donor.

**Figure 11:**
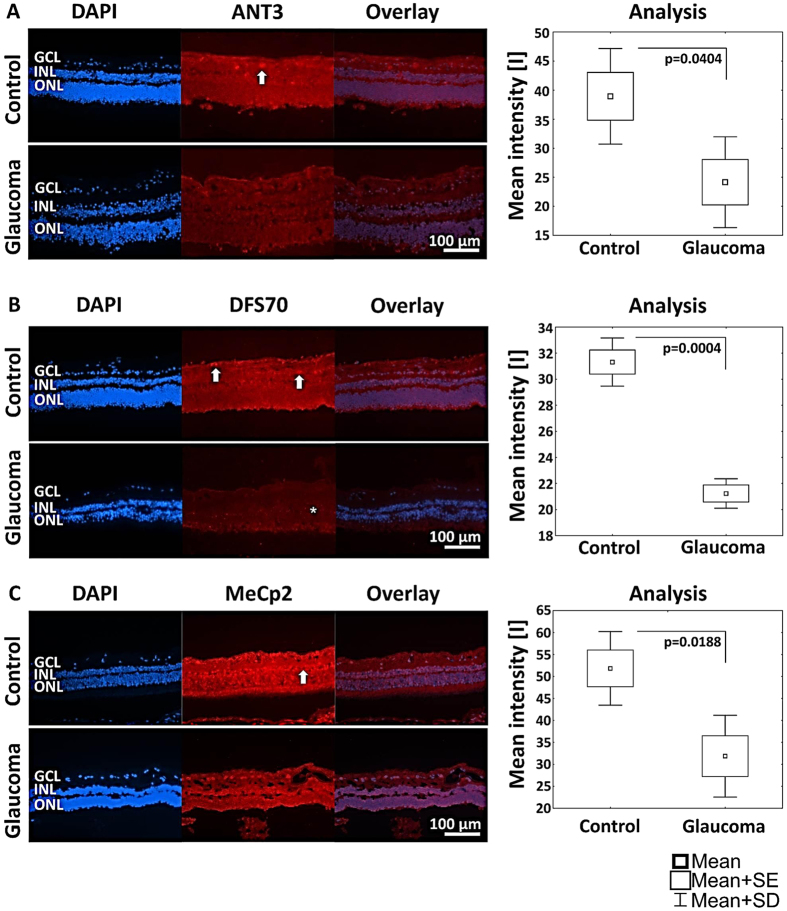
Immunofluorescence was detected and analyzed against candidates ANT3, DFS70 and MeCp2 in control and glaucomatous retinal tissue supporting significantly lower abundance of candidates in glaucomatous retinae compared to control retinae. (**A**) ANT3 shows depositions in the ganglion cell layer (GCL; indicated by arrow). (**B**) DFS70 is distributed in the entire retina with accumulations in the GCL. The distribution of DFS70 in glaucomatous retinal tissue seems to be accumulated in the inner and outer nuclear layer (INL and ONL; highlighted by asterisk). (**C**) MeCp2 shows a strong immunofluorescence in the entire retina with accumulations in the GCL and the INL (indicated by arrow).

**Figure 12:**
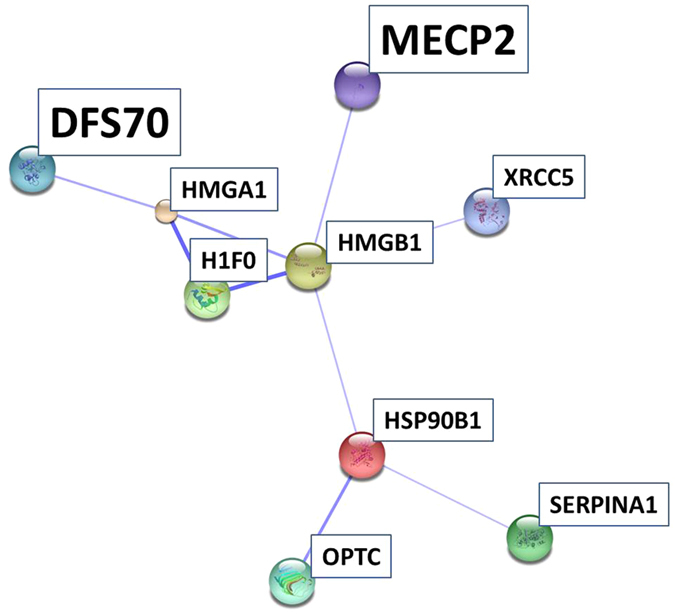
STRING analysis leads to isolation of an interaction network between glaucoma associated candidates. MECP2 and DFS70 are two highlighted members of the interaction network emphasizing their potential role in the disease.

Individual human retina samples (Glaucoma, *N* = 5; Control, *N* = 5) showed a high pattern congruency regarding 1D-SDS-PAGE with the exception of glaucoma donor #*G3,* which displayed an exclusively distinct protein cluster between ~15 and 20 kDa (Fig. 1), primarily referring to exorbitant crystalline upregulation. Due to the risk of masking of low abundant effects glaucoma donor #G3 was excluded from quantitative statistical analysis. However, some samples generated more background than others without affecting the proteomic pattern. BU LC ESI MS analysis of human retina extracts resulted in highly qualitative total ion current (TIC) chromatograms displaying proper peptide elution within 50 min HPLC gradient period leading to accurate protein identification (Fig. 2). Thereby, a sensitive view to the human retina proteome could be obtained. In summary, more than 600 retinal proteins were identified with stringent false discovery rate (FDR < 1%; Supplementary information). For qualitative description of the human retina proteome, exemplary photoreceptor specific proteins like interphotoreceptor matrix proteoglycan 1^26^, rod outer segment membrane protein 1^27^ or rhodopsin^28^, but also numerous synaptic neuroproteins, e.g. neuroplastin^29^, synaptogyrin-1^30^, synaptophysin^31^, synaptosomal-associated protein 25^32^ or synaptotagmin-1^33^ could be mapped. Additional retina and optic nerve resident proteins, e.g. astrocytic phosphoprotein PEA-15^34^, neuronal cell adhesion molecule^35^, glial abundant proteins neurocalcin-Σ^36^ and vimentin^37^ could be detected. Beside reported RGC abundant proteins, Thy-1 membrane glycoprotein, tubulin-ß 3 chain and neurofilament light polypeptide, the RGC selective marker protein RNA binding protein with multiple splicing (RBPMS)^38^,^39^ could not be confirmed in human retinal samples in this work. In contrast, neuronal relevant retinal proteins, non-POU domain-containing octamer-binding protein^40^ and glial fibrillary acidic protein (GFAP)^41^, could be recovered. Therefore, analyzed proteins qualitatively represent the functional human retina proteome featuring the ganglion cell layer (GCL). Interestingly, MUC-9 and proline-rich protein 4 representing characteristic ocular surface proteins could be detected in the samples, most likely artificially derived from the ocular surface during sample preparation. Regarding functional analysis, 95% of mapped retinal proteins could be GO annotated and have been primarily revealed as intracellular proteins. Inferred from label-free quantification (LFQ) statistics, approximately 10% of the whole retina proteome showed distinct alterations between glaucoma and healthy samples emphasized by “unique” LFQ attendance, significant group-specific abundance alterations (p < 0.05) or distinct changes by trend (p < 0.1) (Table 1). In particular, altered candidate proteins predominantly represent intracellular, membrane-resided species (Fig. 3), within the nucleus and/or mitochondria (Fig. 4A). Moreover, candidates were dominated by binding and catalytic features and were found to be involved in cellular key processes like development, cellular transport and cell death (Fig. 4B,C). Proteins like V-type proton ATPase 116 kDa subunit A isoform 1 (VPP1) or arrestin-C (cArr) were revealed below the LFQ abundance threshold in glaucoma samples. Three nucleus proteins, methyl-CpG-binding protein 2 (MeCp2), PC4 and SRFS1-interacting protein 1 (DFS70) and 40S ribosomal protein S7, were revealed to be significantly diminished (p < 0.05) in glaucoma retina samples (Table 1). In contrast, nucleus proteins, guanine nucleotide-binding protein G(i) subunit α-2 and histone H1.0, showed strong tendencies of glaucoma associated level decrease (p < 0.1) (Table 1). Beside nucleus proteins, numerous mitochondrial proteins displayed glaucoma associated level decrement, e.g. ADP/ATP translocase 3 (ANT3), pyruvate dehydrogenase component subunit ß (PDHE1-B) and cytochrome C oxidase subunit 7A2 (COX7A2) (Table 1, Fig. 5). In contrast, retinal dehydrogenase 1 (RALDH 1), retinol-binding protein 3 (IRBP) (Fig. 5) and vesicle-associated membrane protein-associated protein B/C, showed significant level enhancement referring to glaucoma (p < 0.05) (Table 1). Also, stress related proteins including crystallins, serotransferrin and glutathione metabolic proteins indicated level elevation in samples of glaucoma subjects (Table 1, Fig. 5). Since Thy-1, an important RGC marker protein, Thy-1 membrane glycoprotein^42^, could exclusively been LFQ substantiated in glaucoma samples (Table 1), Thy-1 recovery was further inspected. Thereby, BU LC MS determination of the dynamic range of Thy-1 using recombinant protein showed a linear correlation between MS abundance and Thy-1 content between 0.15 and 0.015 μg before and after quantification (*R*^2^ > 0.8; p < 0.05). In the range <0.03 μg Thy-1 raw intensities approximate a value of 1^*^E^6^ generating a LFQ zero value during quantification. Moreover, in this low abundant range only one of initial five Thy-1 specific peptides is detectable, which indicate critical detection of Thy-1 <0.03 μg (Fig. 6). Reanalysis of the study samples supported the presence of Thy-1 in both groups; glaucoma and control, based on raw intensities. Thereby, the protein detection corresponded to two Thy-1 characteristic peptides, which abundances could be recorded close to the detection threshold. BU LC MS analysis of a glaucoma and a control pool of remaining sample material supported identification of Thy-1 in glaucoma samples based on one characteristic peptide by MS/MS. Nevertheless, manual inspection of corresponding chromatograms also supported existence of Thy-1 in the control pool indicated by the identical diagnostic reporter peptide, however not leading to MS/MS based identification (Fig. 7). Using Laser capture microdissection (LCM) several proteins proposed to be particularly glaucoma-associated, e.g. MeCp2, VPP1 or ANT3, could be significantly recovered (p < 0.05) from the GCL of porcine retinae utilizing the BU LC ESI MS platform (Fig. 8, Table 1). Representative validation of glaucoma related proteomic changes was realized in pooled in-solution digests of retina lysates by targeted Accurate Inclusion Mass Screening (AIMS) strategy considering limited sample material (Table 1, Fig. 9). Thereby, selection of a five candidate subset including ANT3, MeCp2, DFS70, cArr and HspB5 was based on functional representation (mitochondria, nucleus, stress indication) and/or GCL support as well as on possibility of redetection of diagnostic reporter peptides in in-solution digested samples. Reporter peptides, one for each candidate protein, were preselected based on their unique amino acid sequence (Table 2). Whereas HspB5 and cArr failed for validation, level diminishment of ANT3, DFS70 and MeCp2 could be supported by targeted AIMS analysis in pooled samples (*N* = 4/group) (Fig. 9). Despite material limitation for sufficient MS/MS fragmentation, these unique reporter peptides corresponding to MeCp2, DFS70 and ANT3 could be detected in a human non-glaucoma GCL LCM preparation also providing evidence of their presence in the human GCL (Fig. 10). Regarding immunohistological validation of selected candidates; ANT3, DFS70 and MeCp2; all three candidates were detectable in all examined human retinae. Thereby, significant changes were observed in the overall intensity between control and glaucomatous tissue samples. ANT3 was found to be distributed in large deposits in the GCL, the inner nuclear layer (INL) and in the outer plexiform layer (OPL) and showed levels of 38.94 ± 8.2 I in control samples compared to 24.15 ± 7.8 I in glaucomatous tissue (−38%, p < 0.04) (Fig. 11A). DFS70 occurred throughout the retina with GCL accumulations and was degraded in glaucomatous retina with 21.23 ± 1.13 I compared to 31.31 ± 1.8 I in control retina (−32%, p < 0.001) (Fig. 11B). MeCp2 displaying accumulations in the GCL and INL showed levels of 51.85 ± 8.39 compared to 31.85 ± 9.31 in control tissue (−39%, p < 0.02) (Fig. 11C). Moreover, STRING analysis indicated evidence for strong interaction between MeCp2 and DFS70. Also, their interaction with nucleus proteins, e.g. histone 1, high mobility group proteins (HMGs) and further proteins, e.g. endoplasmin (HSP90B1) and opticin (Fig. 12) could be documented. In summary, a detailed view to the human retina proteome could be achieved providing a subset of candidate proteins showing level alterations in glaucomatous retina samples. Thereby, special evidence for glaucoma correlation could be provided for ANT3, DFS70 and MeCp2 representative for glaucoma related proteomic alterations in human retinae.

## Discussion

By use of a state-of-the-art BU LC ESI approach^43^ we could identify more than 600 proteins with FDR < 1%, incorporating numerous neuronal relevant protein species. Moreover, the proteomic output fits well to prior mammalian retina studies, e.g. reporting 400 proteins to be visualized from rat retina^10^, more than 900 2D gel protein spots^44^,^45^ and more than 800 proteins identified from mouse retina samples^46^,^47^. Recently, Zhang and colleagues catalogued over 3000 retinal proteins from healthy human donors^48^. Nevertheless, proteomic investigations on human retina, especially diseased human retina samples are still rare. Ethen and colleagues identified over 500 proteins in human AMD donor retinae^49^, 72 secretory proteins were reported from human RPE cells^50^ and Yang and collegues reported several hundred proteins from human retina samples^24^. Qualitatively, many of the identified retinal proteins could be supported by proteomic mammalian retina maps of other working groups focusing on rodents^51^,^52^ or primates^53^. Regarding the human retina proteome examined in the present work, most of the revealed proteins could be classified as intracellular species indicating a high recovery of cellular proteins in contrast to secretory or extracellular proteins. The large amount of membrane proteins probably reflects ionization priority of hydrophobic peptides in ESI experiments^54^,^55^. Moreover, since retina samples in the present study were based on processed retina tissue after removing blood vessels and vitreous residuals the proteomic output can be suggested to refer to the “core proteome” of the human retina explaining a number of >600 proteins and limited representation of secretory proteins. Regarding glaucoma affected retinal proteins, numerous candidates could be recovered from the GCL of porcine retinae by LCM analysis. “Unique” LFQ documentation of RGC specific Thy-1 in glaucoma samples was found contrary to reported reduced levels indicative for RGC loss^56^,^57^,^58^. Thy-1 related reanalysis showed that the protein is present in samples of both groups, control and glaucoma in low abundance and could be identified by two specific peptides, which correspond to the detection threshold <0.03 μg. In a further experiment with pooled samples of remaining tissue, Thy-1 could be identified in glaucoma samples by MS/MS analysis. Nevertheless, Thy-1 could be indirectly affirmed for the control group by reporter peak detection. In consequence of the low abundant recovery of Thy-1 in the study samples, Thy-1 quantification has to be viewed critical. This is a clear study limitation and has to be considered for future experimental designs. However, the detection of Thy-1, even in low abundance supports RGC representation in retinal samples. Moreover, regarding the majority of altered proteins, in first instance nucleus and mitochondrial species were found affected and could be verified to be involved in cellular key processes, e.g. transport, system development and cell death. Mitochondrial dysfunction, involvement in cell death and associated protein alteration has been intensively studied in neurodegeneration^59^ including glaucoma^60^,^61^. In accordance, diminishment of retinal mitochondrial proteins has been documented for neurodegenerative processes, e.g. in a Parkinson monkey model^62^. Confidently, ocular levels of COX7A2, decreased in glaucoma retina samples in the present study, were found to be reduced in glaucomatous primates^63^ and are involved in mitochondrial stress in POAG^64^. In this work, levels of mitochondrial ANT3 were clearly found diminished in glaucomatous retinae demonstrated by MS and immunohistology results. Moreover, ANT3 localization could be shown for the GCL of porcine and human retina fitting well with its documented high abundance in CNS neurons (www.proteinatlas.org). Lately, ANT was found to be part of the mitochondrial ATP synthasome^65^ emphasizing its role for cellular energy supply. In fact, level elevation of ANT3 was reported to enhance cell survival^66^ supporting its key function in glaucomatous neurodegeneration. Level declines of mitochondrial key enzymes ANT3, COX7A2, PDHE1-B and VPP1 support the entanglement of the cellular energy supply system in glaucoma^60^. Confidently, increase of ANT expression and activity has been shown to reduce cellular oxidative stress and apoptosis^67^. Furthermore, ANT3 interactions have been documented for secretogranin-1 (BiomarkerDigger: www.biomarkerdigger.org; Hit Predict: hintdb.hgc.jp), which was demonstrated to play a key role in Parkinson disease^68^. Also, interactions were reported between ANT3 and members of the tumor necrosis factor (TNF) receptor superfamily (Hit Predict: hintdb.hgc.jp) involved in glaucomatous neurodegeneration^69^. Beside mitochondrial protein alterations, levels of certain cytoplasmic proteins were found to be altered in glaucoma, e.g. cArr, which has been documented to be involved in glaucoma photoreceptor degeneration^70^ and found to be diminished in AMD macula^71^. Interestingly, anti-cArr auto-antibodies have been detected in multiple sclerosis^72^ supporting cArr association to neurodegenerative processes. However, even if cArr could not been validated in this work, it needs to be considered for future prospects. Additionally, further proteomic alterations can be associated to neurodegenerative processes in the retina. Glaucoma related level decrease of protein NipSnap homolog 3A is in confidence with downregulation of NipSnap proteins in Alzheimer disease^73^ and hyperoxia/erythropoietin stressed brain tissue^74^. NipSnap 3 was reported to be involved in apoptosis via antagonizing x-linked inhibitor of apoptosis protein (XIAP) caspase 3 inhibition^75^. A comparable role was proposed for nucleus proteins, heterogeneous nuclear ribonucleoproteins (hnRPs) C1 and C2^76^. In accordance, a key role of hnRNP A1 in neurodegeneration was highlighted^77^. Confidently, the observed decline of hnRNP Q and U most likely refer to neuronal damages in glaucoma retinae. Diminishment of MeCp2, a retinal^48^ and neuron expressed nucleus key trigger protein in Rett syndrome^78^,^79^ fits well with its high CNS expression levels, turnover, histone modification, DNA and protein binding features as well as its level declines observed in neurodegenerative diseases^80^. Beside the neuronal key regulator function of MeCp2 in gene expression^81^, MeCp2 deficiency was suggested to affect directly the neuronal cytoskeleton^82^ triggering nucleus stability, proliferation and cell viability^83^. Since there is growing evidence of MeCp2 participation in neurodegenerative pathologies^84^, Bogdanovic & Veenstra proposed a “well-balanced requirement of MeCp2 levels for proper function of the mammalian CNS”^85^, most likely encircling retinal health. Despite its high abundance in neuronal cells, which is in confidence with the supported MeCp2 retinal GCL localization in the present work, it is not known why MeCp2 level diversification exclusively affect neuronal cells^86^. Also, its high expression level and its associated role in the retina remains controversial^87^. From a functional view MeCp2 has been demonstrated to display multiple binding features to several proteins^80^, e.g. to methyl-cytosine binding domain protein 2 (MBD2)^88^ or neurological relevant proteins like α-thalassemia/mental retardation, X-linked protein (ATRX)^89^ and γ-synuclein^90^, which underlines its potential to modulate molecular pathways in the course of neurodegeneration. Especially, MeCp2 DNA binding and neuronal gene expression regulating features^91^ give new perspective to neurodegenerative processes in the glaucomatous retina. Interestingly, DFS70 displaying glaucoma related decline, shares functional similarities to MeCp2, e.g. nucleus residence and DNA- and protein binding properties (UniProt: www.uniprot.org), suggestible for neurodegenerative involvement. Moreover, STRING interaction analysis supported strong association of MeCp2 and DFS70, both detected in the retina GCL in the present work, in confidence with their high abundance in CNS neurons (www.proteinatlas.org). Consistently, Leoh and colleagues documented direct binding of MECP2 to DFS70^92^. Misregulation of MeCp2, histone H1 and HMGs were suggested indicative for chromatin composition changes in mouse myoblasts^93^ and could reflect neurodegenerative related chromatin alterations^94^. Regarding MeCp2/HMGB1 interaction^95^, the existence of a chromatin-binding protein interaction complex affected in the glaucomatous retina is thinkable. However, the exact role of these chromatin-binding proteins has not been uncovered so far and needs to be addressed in future studies, e.g. in glaucoma animal models. Also, an indirect interaction between chromatin-binding proteins to glaucoma related proteins, opticin^96^ and endoplasmin (HSP90B1)^97^, could be observed. Beside elevated endoplasmin levels, increased levels of further characteristic stress marker proteins could be detected in glaucomatous retinae, e.g. glutathione synthetase associated to the glutathione system involved in neurodegeneration^98^,^99^. Furthermore, glutathione S-transferase (GST) has been described as a glaucoma associated stress marker^100^ and increased serum GST immunoreactivity has been documented in glaucoma^101^. In fact, various GST species were found to be implemented to degenerative stress related pathologies^102^,^103^,^104^,^105^. Increase of serotransferrin as an acute phase protein^106^ fits well with differential expression of the protein observed in serum of glaucoma patients^107^. Also, indicative alterations of crystallins^51^,^108^ have been documented for diabetic retinopathy^109^,^110^ and glaucoma^111^ and especially upregulation in retinal disorders have been reviewed^112^. Retina associated protective potential has been discussed for crystallins^113^ and retinol dehydrogenase^114^, probably explaining the observed enhancements of these proteins. Exorbitant increase of crystallins was observed in the excluded glaucoma subject #*G3* and consequently other stress factors beyond neurodegenerative processes cannot be precluded for crystalline increment. Decrease of 6-phosphofructokinase levels observed in glaucoma retinae is in confidence with downregulation in neurodegenerative Alzheimer^115^ and Down syndrome brains^116^ and supports energy metabolism implementation. Increase of α-1-antitrypsin could be indicative for elevated autoreactivity for this protein observed in serum of glaucoma patients demonstrated by our group^117^. Moreover, increase of the protein is in confidence with overexpression in serum of newly recruited glaucoma patients^107^ and α-1-antitrypsin is suggestive to play a role in Alzheimer´s disease^118^. In conclusion, based on proteomic data three functional classes could be supported to be involved in glaucomatous processes: mitochondrial, stress and nucleus proteins indicating an impairment of energy metabolism, stress response and gene expression alterations in the course of retinal neurodegenerative processes. Numerous proteins could be supported to be GCL expressed revealed by LCM, underlining their involvement at the site of damage. However, the present study is limited by the number of donor retinae and sample material. Another limitation is the focus on progressed glaucoma and use of postmortem retinal material. Accordingly, the role of mitochondrial proteins like ANT3 and the function of nucleus proteins, especially hnRNPs, DFS70 and MeCp2 in the course of neurodegeneration need to be in the focus of future projects determining accurate candidate protein dynamics during glaucoma progression. In conclusion, the present work provides a sensitive image of the human “retina core proteome” supporting mitochondrial and nucleus proteome entanglement of glaucomatous neurodegeneration and highlights new molecular players, e.g. ANT3, DFS70 and MeCp2 to be considered in future glaucoma studies.

## Methods

### Patients and samples

Donor eyes were provided by the Cornea Bank of Rhineland-Palatine, Department of Ophthalmology, University Medical Center Mainz, Germany. Organ donations followed the guidelines for corneal donations regulated by the German Transplantation Law in agreement with the requirements of the Declaration of Helsinki. The use of remaining ocular donor tissue not required for transplantation for study purposes has been explicitly approved by the relatives of the deceased and the local ethics committee. Retina tissue samples were prepared from post-mortem donor eyes (right or left eye). Glaucoma donors (*N* = 5; 3 females, 2 males) were 86 +/−9 years old, control donors (*N* = 5; 3 females, 2 males) were 82 +/−7 years old. For LCM experiments an additional eye bulb of a 69-year old female non-glaucoma donor was used. Donor exclusion criteria were defined as the following: any kind of ocular diseases including infection and tumors, systemic infections (viral, fungal, bacterial) incl. hepatitis, HIV, malignat neoplasia, autoimmune diseases including multiple sclerosis, Creutzfeldt-Jakob, Parkinson, Alzheimer, amyotrophic lateral sclerosis as well as need of dialysis. Ophthalmologic background and clinical history were requested from closest relatives, general practitioner and last attending doctor. Clinical phenotype of glaucoma could not be defined in donors, which is a distinct limitation of the present study. Retina tissue was extracted from eye cups (sagittal half of bulbs) and isolated from pigment epithelium, ciliar body, blood vessels and vitreous residuals in phosphate buffered saline under a binocular microscope. Liquid nitrogen snap frozen retina tissue samples were grinded by mortar and pestle, added 0.1% SDS, mixed for 20 min at room temperature and centrifuged at 14000 rpm at 4 °C. Supernatants were used for total protein measurement using the Pierce BCA Protein Assay Kit (Thermo Scientific, Rockford, USA) following the manual of the manufacturer.

### BU LC ESI MS

Retina samples (Glaucoma, *N* = 5; Control, *N* = 5; 40 μg/sample) were run on 12% Bis-Tris 10-well prepared SDS PAGE minigels under reducing condition at 150 V followed by Coomassie Brilliant Blue staining (Invitrogen, Carlsbad, CA, USA). Sample lanes (individual and pools) were divided into 17 slices/lane followed by trypsin digestion using a modified protocol of Shevchenko *et al*.^119^. Dehydrated gel pieces were soaked with 100 μl of sequencing grade modified trypsin (Promega Corporation, Madison, WI, USA) solution [15 ng/μl] and incubated overnight at 37 °C. Lyophilized peptides were double purified by ZIPTIP C18 solid phase extraction (Millipore, Billerica, MA, USA) prior to MS analysis according to the protocol of the manufactor following lyophilization and storage −20 °C prior to MS analysis. Resolubilized peptides (20 μl 0.1% TFA/slice digest) were analyzed by use of LC ESI MS. The LC system consisted of a Rheos Allegro pump (Thermo Scientific, Rockford, IL, USA) downscaled to a capillary system (6.7+/− 0.03 μl/min flow rate) as already described with weak modifications (21, 22). The column system consisting of a 30 × 0.5 mm BioBasic C18 coupled to a 150 × 0.5 mm BioBasic C18 analytical column (Thermo Scientific, Rockford, IL, USA) and protected by an A316 online precolumn filter (Upchurch Scientific, Washington, DC, USA) was run under gradient elution (buffers: A = 98% H_2_O, 1.94% ACN, 0.06% methanol, 0.05% formic acid; B = 95% ACN, 3% methanol, 2% H_2_O, 0.05% formic acid). The 50 min gradient was programmed as follows: 15–20% B (0–2 min), 20–60% B (2–35 min), 60–100% B (35–40 min), 100–0% B (40–45 min), 0% B (45–50 min). The LC system was directly connected to the ESI source of a hybrid linear ion trap (LTQ) Orbitrap (FT) XL instrument (Thermo Fisher Scientific, Rockford, IL, USA) introducing ions by use of a low flow metal needle (Thermo Fisher Scientific, Rockford, IL, USA). 5 μl of peptides were injected for each run by use of a PAL HTS robot (CTC Analytics, Zwingen, Switzerland). To avoid sample carryover effects 30 min washing runs injecting 80% ACN to the system were realized. The whole workflow was administered by XCalibur 2.0.7 SP1 (Thermo Fisher Scientific Rockford, IL, USA). For FT MS measurements a range of 300–2000 m/z in the instruments positive mode was selected and the following parameters were adjusted: max. injection time =50 ms (LTQ), 500 ms (FT), activation = collision induced decay (CID), normalized collision energy  = 35, activation time = 30 ms, activation Q = 0.25. The top five centroid detected monoisotopic peaks (charge state = 1+, 2+, 3+, 4+; intensity > 500) were selected within an isolation width of 2 m/z for fragmentation in each scan event considering dynamic exclusion of 90 s, repeat duration of 30 s and resolution of 30000.

### Protein identification and quantification

Combined LC MS raw files were transferred to MAXQuant version 1.4.1.2 (Max Planck Institute of Biochemistry, Martinsried, Germany; www.maxquant.org) and its built-in Andromeda search engine for protein identification and quantification. MS/MS fragment spectra were search against the SwissProt human database (SwissProt_111101, 533049 sequences, 189064225 residues). Thereby, settings were set as follows: peptide mass tolerance =±30 ppm, fragment mass tolerance =±0.5 Da, fixed modification = carbamidomethylation (C), variable modifications: oxidation (M), acetylation (protein N-term), enzyme = trypsin, allowed missed cleavages: 2. Proteins were identified with a false discovery rate (FDR) <1% with ≥6 amino acid residues and only ‘unique plus razor peptides’ that belong to a protein, which was also included for quantification. LFQ normalized peak intensities^120^ were transferred to Statistica (Version 10, Statsoft, Tulsa, OK, USA) for group specific protein level comparison. Protein LFQ normalized peak intensities of glaucoma and control subjects were analyzed using t-test except donor sample #*G3,* which was excluded from analysis due to its deviated gel pattern. Proteins displaying significant group-specific level alterations (p < 0.05) or distinct tendencies (p < 0.1) were assigned candidates. For LCM analysis human as well as porcine RGC based MS raw files were combined and matched against the SwissProt human database using Proteome Discoverer version 1.1 (Thermo Scientific, Rockford, IL, USA) and MASCOT version 1.4.1.2 using a more relaxed strategy based on a 95% probability identification threshold.

### Functional analysis (GO and interaction analysis)

Candidate proteins were inspected by gene ontology (GO) analysis using Cytoscape version 2.8.3 with integrated BINGO 2.44 plugin (www.cytoscape.org). Interaction analysis was realized by use of STRING version 10 (http://string-db.org)^121^ considering a medium confidence score of 0.4 for interactions. Moreover particular candidates were manually searched for localization and interaction using online databases (www.proteinatlas.org, www.biomarkerdigger.org, Hit Predict: hintdb.hgc.jp).

### Targeted MS

Validation of a selected candidate protein subset was realized by an already established in-solution tryptic digestion/LC ESI MS protocol^122^ focusing on pooled samples for proper protein amount (10 μg/subject; 40 μg/group, *N* = 4/group, *N* = 4 technical replicates) following Accurate Inclusion Mass Screening (AIMS) strategy based on quantification of unique reporter peptides^123^, each representing one particular candidate protein. Selection of these peptides for the inclusion list was carried out manually based on the msms.txt file resulting from MaxQuant analysis. For inclusion list-dependent acquisition the use of global parent list was enabled, the dynamic exclusion segment was disabled and the m/z tolerance around targeted precursors was set ±10 ppm. The acquired MS spectra were analyzed by MaxQuant for peptide and protein identification. Extracted spectra were searched against Uniprot/Swissprot human database using settings with peptide mass tolerance of ±10 ppm, fragment mass tolerance of ±0.5, peptide charge state of +2 with FDR < 1% for identification. The output of the generated ‘peptides.txt’ from MaxQuant were exported in an excel workbook format (Microsoft Office Excel 2010) and the sum absolute intensity of the signature peptides for each proteins were transferred to Statistica for unpaired t-test analysis (p < 0.05).

### Laser-capture microdissection (LCM)

Due to their retina model comparability^124^ freshly enucleated porcine (*Sus scrofa*) eye bulbs (*N* = 3) obtained from the local slaughterhouse (Robert-Bosch-Str. 23–25, 55232 Alzey, Germany) were embedded in Tissue-Tek O.C.T. compound (Sakura Finetek, Tokyo, Japan), deep frozen in liquid nitrogen and sliced at −27 °C to 30 μm transversal sections using a CM 1850 cryotome (Leica Biosystems Nussloch GmbH, Nussloch, Germany). Slices were collected on UV-pretreated PEN membrane slides and transferred to cool 70% ethanol, stained with hematoxylin for 1 min and dehydrated with 70%, 96% and 100% ethanol for 2 min. RGC tissue LCM was realized by use of a PALM MicroBeam Laser system (Zeiss, Oberkochen, Germany) using 5x magnification. For each LCM extract a RGC layer tissue area on average of 5.3 mm^2^ has been dissected approximately representing 19950 cells. Single cutouts had a size of 0.024 mm^2^ and were captured in AdhesiveCap 500 opaque caps (Carl Zeiss Jena GmbH, Jena, Germany) under RoboLPC laser control. Tissue preparations were extracted by shaking using a KS250 Basic Intellimixer (Kika Labortechnik, Cologne, Germany) at 25 rpm and 4 °C for 2.5 h. Suspensions were transferred to BU LC ESI MS as already described. Finally, to recover candidates in human GCL, the above described LCM BU LC ESI MS workflow was applied on 30 μm transversal sections of a single non-glaucoma donor eye bulb approximately recovering 15500 cells. Corresponding LC MS TIC chromatograms were screened for the occurrence of selected candidate (DFS70, ANT3 and MeCp2) unique reporter peptides to indicate their existence in the human retina GCL.

### Immunodetection of DFS70, ANT3 and MeCp2 in retinal cross sections of healthy and glaucomatous humans

Retina specimen from sagittally split bulbi of age and gender matched donor eyes (N = 4 glaucoma, N = 4 control) corresponding to the proteomic analysis were fixed in 4% formaldehyde (Carl Roth, Karlsruhe, Germany) for 10 days before paraffin embedding by use of an automated Tissue-Tek Auto TEC embedder (Sakura Finetek, Tokyo, Japan) applying the following steps: 1. fixation, 2. dehydration, 3. clearing and 4. paraffination. For each patient, four serial cross sections with 5 μm thickness were sliced on a 2030 Biocutrotation microtome (Reichert &Jung), transferred onto Superfrost plus slides (Thermo Scientific, Schwerte, Germany), dried and stored at 37 °C. For staining slices were dewaxed in xylene three times and rehydrated in a sequential ethanol gradient (100%, 100%, 96%, 96%, 70%, each 5 min) to Aqua dest. Antigen retrieval was performed in preheated Target Retrieval Solution (DAKO, Hamburg, Germany) at 87 °C for 30 min, followed by 20 min at room temperature before short washes in PBS (Dulbecco’s PBS, Sigma Aldrich, St. Louis, MO, USA). Slices were blocked with 1% normal donkey serum/0.5% bovine serum albumin/PBS for DFS70 staining or with 3% normal goat serum/0.1%TritonX/PBS for ANT3 and MeCp2 staining for 30 min. Primary antibodies were diluted 1:200 and incubated at 4 °C overnight. Spare antibodies were washed off using PBS twice for 15 min on a rotating platform. All secondary antibodies (ANT3: Ref. orb101369, Biorbyt Ltd., Cambridge, UK; DFS70: Ref. sc-33371, Santa Cruz Biotechnology, Dallas, TX, USA; MeCp2: Ref. ChIP Grade ab2828, Abcam, Cambridge, UK) were diluted 1:200 in PBS and incubated on tissue for 60 min. Again, spare antibodies were washed off before tissue was mounted using diamidin-2-phenylindol (DAPI) supplemented Vectashield (Vector, Burlingame, CA, USA). Negative controls were conducted through incubation of equally treated slides with secondary antibodies exclusively. For quantification mounted tissue was photographed immediately after staining using Eclipse TS 100 microscope with a DS-Fi1-U2 digital microscope camera and an ELWD 20x/0.45 S Plan Flour Ph1 ADM objective and recorded by the imaging software NIS Elements version 4.10 64 bit (microscope, camera, equipment and software were purchased from Nikon, Tokyo, Japan). Exposure time settings were the same for samples from healthy and glaucomatous patients. At least 30 pictures were taken per patient for analysis. Quantification of intensity differences was performed using Image J (rsb.info.nih.gov/ij, NIH, Bethesda, MD, USA) and statistical differences in the overall intensity (I) were calculated by using Breakdown ANOVA Test and unpaired t-test in Statistica.

### Focused Thy-1 analysis

Dynamic range of recombinant human Thy-1 (P01, Ref. H00007070-P01, Abnova, Heidelberg, Germany) was determined by use of the established BU LC MS workflow encircling 1D-SDS PAGE and LC MS. At first, three identical sample dilution subsets of Thy-1 were run on three gels allowing triplicate analysis of each Thy-1 dilution in the LC MS system. In-gel digestion and ZIP TIP purification of Thy-1 dilution samples were carried out as already described. Accordingly, triplicate samples of 0.15, 0.12, 0.09, 0.03, 0.015 μg Thy-1 were analyzed by the LC MS system following MaxQuant analysis considering MaxQuant Raw and LFQ data acquisition. Mean MaxQuant Raw and LFQ intensity values of Thy-1 triplicate LC MS measurements were transferred to Statistica (version 10, Statsoft, Tulsa, OK, USA) for dynamic range determination by linear regression analysis. Furthermore, for validation of Thy-1 regulation, LC MS TIC raw files corresponding to Thy-1 SDS PAGE mass migration area of the control and the glaucoma sample pool (40 μg/lane) were analyzed by ProteomeDiscoverer and manually inspected for occurrence of Thy-1 specific unique peptide corresponding to 856.46 *m/z*.

## Additional Information

**How to cite this article**: Funke, S. *et al*. Glaucoma related Proteomic Alterations in Human Retina Samples. *Sci. Rep.* **6**, 29759; doi: 10.1038/srep29759 (2016).

## Supplementary Material

Supplementary Information

## Figures and Tables

**Table 1:** Human candidate retinal proteins (FDR <1%) that were A1. documented exclusively in the control group based on zero LFQ values in the glaucoma group and therefore named “unique”; A2. significantly (p < 0.05) or distinctly (p < 0.1) decreased in the glaucoma group; B1. documented exclusively in the glaucoma group based on zero LFQ values in the control group and therefore named “unique”; B2. significantly (p < 0.05) or distinctly (p < 0.1) increased in the glaucoma group.

Protein name	Gene name	Entry	Entry name			LCM
**A1: “Unique” control retinal proteins**						
V-type proton ATPase 116 kDa subunit a isoform 1 (VPP1)	ATP6V0A1	B7Z641	B7Z641_HUMAN			√
Arrestin-C (cArr)	ARR3	P36575	ARRC_HUMAN			
Protein NipSnap homolog 3A	NIPSNAP3A	Q9UFN0	NPS3A_HUMAN			
Heterogeneous nuclear ribonucleoprotein Q (hnRPQ)	SYNCRIP	F6UXX1	F6UXX1_HUMAN			
6-phosphofructokinase, liver type	PFKL	P17858	K6PL_HUMAN			
Elongation factor Tu, mitochondrial	TUFM	P49411	EFTU_HUMAN			
40S ribosomal protein S19	RPS19	P39019	RS19_HUMAN			√
Peripherin-2	PRPH2	P23942	PRPH2_HUMAN			
Protein SET	SET	Q01105	SET_HUMAN			
Reticulocalbin-2	RCN2	Q14257	RCN2_HUMAN			
**A2: Glaucoma decreased retinal proteins**				**Glaucoma/Control**	**p-value**	
Calcium-binding mitochondrial carrier protein Aralar2	SLC25A13	Q9UJS0	CMC2_HUMAN	0,15	0,0060	
Methyl-CpG-binding protein 2 (MeCp2)	MECP2	P51608	MECP2_HUMAN	0,15	0,0117	√
NADH dehydrogenase [ubiquinone] 1 ß subcomplex sub. 9 (CI-B22)	NDUFB9	Q9Y6M9	NDUB9_HUMAN	0,15	0,0157	
40S ribosomal protein S7	RPS7	P62081	RS7_HUMAN	0,17	0,0748	
3-hydroxyacyl-CoA dehydrogenase type-2	HSD17B10	Q99714	HCD2_HUMAN	0,18	0,0357	
Trifunctional enzyme subunit α, mitochondrial	HADHA	P40939	ECHA_HUMAN	0,19	0,0498	
Myosin-11/9	MYH11	P35749	MYH11_HUMAN	0,21	0,0968	
60S acidic ribosomal protein P0; P0-like	RPLP0	P05388	RLA0_HUMAN	0,27	0,0238	√
Pyruvate dehydrogenase E1 comp. sub. ß, mitochondrial (PDHE1-B)	PDHB	P11177	ODPB_HUMAN	0,31	0,0274	√
High mobility group protein HMG-I/HMG-Y	HMGA1	P17096	HMGA1_HUMAN	0,40	0,0239	
Small nuclear ribonucleoprotein Sm D3	SNRPD3	P62318	SMD3_HUMAN	0,43	0,0109	
(ATP-dependent) 6-phosphofructokinase, muscle type	PFKM	P08237	PFKAM_HUMAN	0,47	0,0743	√
Vesicle-fusing ATPase	NSF	P46459	NSF_HUMAN	0,49	0,0657	√
Heterogeneous nuclear ribonucleoprotein L	HNRNPL	P14866	HNRPL_HUMAN	0,51	0,0777	
PC4 and SFRS1-interacting protein (DFS70)	PSIP1	O75475	PSIP1_HUMAN	0,56	0,0271	
Cytochrome c oxidase subunit 7A2, mitochondrial (COX7A2)	COX7A2	P14406	CX7A2_HUMAN	0,58	0,0893	
ADP/ATP translocase 3 (ANT3)	SLC25A6	P12236	ADT3_HUMAN	0,61	0,0199	√
Ras-related protein Rab-11A; B	RAB11A	Q15907	RB11B_HUMAN	0,62	0,0174	
Heterogeneous nuclear ribonucleoprotein U	HNRNPU	Q00839	HNRPU_HUMAN	0,62	0,0494	
Histone H1.0	H1F0	P07305	H10_HUMAN	0,67	0,0729	
Putative elongation factor 1-α-2;1; like 3	EEF1A1P5	Q5VTE0	EF1A3_HUMAN	0,69	0,0148	√
Guanine nucleotide-binding protein G(i) subunit α-2	GNAI2	P04899	GNAI2_HUMAN	0,69	0,0930	√
**B1: “Unique” glaucoma retinal proteins**						
Protein name	Gene name	Entry	Entry name			LCM
Glutathione synthetase	GSS	P48637	GSHB_HUMAN			
Septin-8	SEPT8	Q92599	SEPT8_HUMAN			
Opticin	OPTC	Q9UBM4	OPT_HUMAN			
Calcium-binding mitochondrial carrier protein Aralar1	SLC25A12	O75746	CMC1_HUMAN			√
Serpin B6	SERPINB6	P35237	SPB6_HUMAN			
Neuroplastin	NPTN	Q9Y639	NPTN_HUMAN			
Carbonyl reductase [NADPH] 1	CBR1	P16152	CBR1_HUMAN			
4-trimethylaminobutyraldehyde dehydrogenase	ALDH9A1	B4DXY7	B4DXY7_HUMAN			
ß-crystallin A3;isoform A1/Σ4/Σ7/Σ8	CRYBA1	P05813	CRBA1_HUMAN			
Ig γ-2 chain C region	IGHG2	P01859	IGHG2_HUMAN			
ß-crystallin B1	CRYBB1	P53674	CRBB1_HUMAN			
Phakinin	BFSP2	Q13515	BFSP2_HUMAN			
α-1-antitrypsin;Short peptide from AAT	SERPINA1	P01009	A1AT_HUMAN			
Sideroflexin-1	SFXN1	Q9H9B4	SFXN1_HUMAN			√
X-ray repair cross-complementing protein 5	XRCC5	P13010	XRCC5_HUMAN			
N(G), N(G)-dimethylarginine dimethylaminohydrolase 1	DDAH1	B4DGT0	B4DGT0_HUMAN			
Hippocalcin-like protein 1	HPCAL1	P37235	HPCL1_HUMAN			
Thy-1 membrane glycoprotein	THY1	J3QRJ3	J3QRJ3_HUMAN			
V-type proton ATPase subunit G 1/G2	ATP6V1G1	O75348	VATG1_HUMAN			
Cytosolic non-specific dipeptidase	CNDP2	Q96KP4	CNDP2_HUMAN			
**B2: Glaucoma increased retinal proteins**				**Glaucoma/Control**	**p-value**	
Retinal dehydrogenase 1 (RALDH 1)	ALDH1A1	P00352	AL1A1_HUMAN	11,73	0,0080	
High mobility group protein B1; Putative B1-like 1	HMGB1	Q5T7C4	Q5T7C4_HUMAN	7,39	0,0745	
Vesicle-associated membrane protein-associated protein B/C	VAPB	O95292	VAPB_HUMAN	6,51	0,0023	√
Annexin A2; Putative A2-like	ANXA2	P07355;	ANXA2_HUMAN	4,81	0,0962	
Reticulon-3	RTN3	O95197-3	RTN3_HUMAN	3,69	0,0922	√
Glutathione S-transferase Mu 3 (hGSTM3-3)	GSTM3	P21266	GSTM3_HUMAN	3,61	0,0529	
Dihydropteridine reductase	QDPR	P09417	DHPR_HUMAN	2,37	0,0950	
Retinol-binding protein 3 (IRBP)	RBP3	P10745	RET3_HUMAN	2,12	0,0427	
α-crystallin B chain (HspB5)	CRYAB	P02511	CRYAB_HUMAN	1,66	0,0599	√
Endoplasmin	HSP90B1	P14625	ENPL_HUMAN	1,58	0,0575	√
Serotransferrin	TF	P02787	ENPL_HUMAN	1,46	0,0872	√
Rab GDP dissociation inhibitor α	GDI1	P31150	ENPL_HUMAN	1,40	0,0541	

In case of multiple isoforms, only the first gene, entry accession and entry name are given. In case of candidate recovery in the porcine retinal GCL using LCM, candidates are highlighted by check mark in the LCM column. ANT3, MECP2 and DFS70 presence was supported by MS detection of diagnostic *m/z* peaks in human LCM preparation (illustrated in Fig. 10, not highlighted in the table).

**Table 2:** Subset of representative human retinal candidate proteins selected for AIMS validation in retina sample pools.

Protein	Gene names	Mass [Da]	m/z	Amino acid sequence	Charges	Glaucoma/Control	Valid
ADP/ATP translocase 3 (ANT3)	SLC25A6	1232,68	617,34	DFLAGGIAAAISK	2	0,38	√
PC4 and SFRS1-interacting protein (DFS70)	PSIP1	1631,76	816,88	GFNEGLWEIDNNPK	2	0,80	√
Methyl-CpG-binding protein 2 (MeCp2)	MECP2	1954,89	978,45	VGDTSLDPNDFDFTVTGR	2	0,65	√
α-crystallin B chain (HspB5)	CRYAB	1164,65	583,33	VLGDVIEVHGK	2	1,08	×
Arrestin-C (cArr)	ARR3	1821,94	911,97	VQFAPPEAGPGPSAQTIR	2	0,99	×

Analysis of technical replicate runs (*N* = 4; *N* = 4 individuals/pool sample) supported glaucoma related significant level diminishment of ANT3, MeCp2 and distinct decline of DFS70 highlighted by check mark in the column.
